# Delta neutrophil index as a predictive and prognostic factor for Candidemia patients: a matched case-control study

**DOI:** 10.1186/s12879-020-05117-0

**Published:** 2020-06-05

**Authors:** So Yeon Park, Jin Seo Lee, Jihyu Oh, Ji-Young Park

**Affiliations:** 1 Division of Infectious Diseases, Kangdong Sacred Heart Hospital, Hallym University School of Medicine, 150 Seongan-ro, Gangdong-gu, Seoul, 134-701 Republic of Korea; 2 Department of Laboratory Medicine, Kangdong Sacred Heart Hospital, Seoul, South Korea

**Keywords:** Candidemia, Delta-neutrophil index (DNI), Predictor, Prognostic factor

## Abstract

**Background:**

Delayed antifungal therapy for candidemia leads to increased mortality. Differentiating bacterial infection from candidemia in systemic inflammatory response syndrome (SIRS) patients is complex and difficult. The Delta Neutrophil Index (DNI) has recently been considered a new factor to distinguish infections from non-infections and predict the severity of sepsis. We aimed to assess if the DNI can predict and provide a prognosis for candidemia in SIRS patients.

**Methods:**

A matched case-control study was conducted from July 2016 to June 2017 at Kangdong Sacred Heart Hospital. Among patients with a comorbidity of SIRS, those with candidemia were classified as the case group, whereas those with negative blood culture results were classified as the control group. The matching conditions included age, blood culture date, and SIRS onset location. Multivariate logistic regression was performed to evaluate DNI as a predictive and prognostic factor for candidemia.

**Results:**

The 140 included patients were assigned to each group in a 1:1 ratio. The DNI_D1 values measured on the blood culture date were higher in the case group than in the control group (*p* <  0.001). The results of multivariate analyses confirmed DNI_D1 (odds ratio [ORs] 2.138, 95% confidential interval [CI] 1.421–3.217, *p* <  0.001) and *Candida* colonization as predictive factors for candidemia. The cutoff value of DNI for predicting candidemia was 2.75%. The area under the curve for the DNI value was 0.804 (95% CI, 0.719–0.890, *p* < 0.001), with a sensitivity and specificity of 72.9 and 78.6%, respectively. Analysis of 14-day mortality in patients with candidemia showed significantly higher DNI_D1 and DNI_48 in the non-survivor group than in the survivor group.

**Conclusions:**

DNI was identified as a predictive factor for candidemia in patients with SIRS and a prognostic factor in predicting 14-day mortality in candidemia patients. DNI, along with clinical patient characteristics, was useful in determining the occurrence of candidemia in patients with SIRS.

## Background

The number of candidemia cases has gradually been increasing due to the development of immunosuppressive treatments and invasive procedures. However, despite advances in medical science, the mortality rate of candidemia remains high, at 35–60% [1]. The primary factor in reducing candidemia-induced mortality rates is the early administration of appropriate antifungal therapy (AAT) [2]. Delayed AAT results in increased mortality. Thus, various markers and testing methods have been developed to predict candidemia to enable early-stage antifungal therapy [3–5]. However, there are cases in which these testing methods cannot be easily applied in actual treatment or are difficult to be commonly used.

Moreover, studies reported varying degrees of sensitivity for the developed clinical scores or testing methods [3, 6]. Several recent studies proposed the delta neutrophil index (DNI) as a promising predictive and prognostic marker for sepsis [7–9]. Thus, we evaluated DNI as a predictive marker of candidemia in patients with systemic inflammatory response syndrome (SIRS) and as a prognostic marker for patients with candidemia.

## Methods

### Study setting and patients

This study was performed at Kangdong Sacred Heart Hospital, a university-affiliated hospital with 640 beds, including 40 beds in three intensive care units (ICUs), located in Seoul, South Korea. The Institutional Review Board of Kangdong Sacred Heart Hospital approved this study (2019–04-003). Informed consent was not required by the board because of the retrospective design of the study. Between July 2016 and June 2018, we performed a retrospective review of patient medical records and microbiology laboratory databases. The case group included patients aged 20 years or older who developed candidemia during the study period. In cases of duplicate candidemia, only the initial episode was enrolled in the study. The matched control group included patients aged 20 years or older displaying SIRS during the same period with negative blood culture results. Case and control patients were matched at a 1:1 ratio using the following matching criteria: 1) blood culture procedure dates within ±3 days, 2) age ± 3 years, and 3) same patient location at SIRS onset (ICU vs. general ward). Patients with hematologic malignancies except for lymphoma, neutropenia, and those who received granulocyte colony-stimulating factor were excluded from this study because these conditions may affect hematologic parameters.

### DNI measurement and other laboratory methods

The blood samples for DNI measurement were transferred to the laboratory department in ethylenediaminetetraacetic acid (EDTA) tubes. The DNI was determined within 1 h of blood sampling.

The DNI is included as part of the routine complete blood count (CBS) test at our hospital. The DNI was calculated using an automatic cell analyzer (ADVIA 2120 Hematology System, Siemens Healthcare Diagnostics, Forchheim, Germany) [10]. This cell analyzer counts white blood cells (WBCs) in independent myeloperoxidase (MPO) and nuclear lobularity channels. The formula for calculating DNI is as follows: DNI (%) = (neutrophil and eosinophil subfractions measured in the MPO channel by a cytochemical MPO reaction)-(polymorphonuclear neutrophil [PMN] subfraction measured in the nuclear lobularity channel by a reflected light beam). Blood cultures were ordered at the discretion of the primary physician based on the presence of the signs and symptoms of SIRS. Each set of blood samples was inoculated into one aerobic and anaerobic bottle and immediately loaded into a BacT/ALERT 3D Microbial Detection System (bioMerieux, Inc., Durham, NC, USA). Candida species identification was done using the automated Vitek 2 Yeast Biochemical Card (bioMerieux, Inc.).

### Study variables and definitions

Clinical data extracted from the patients’ medical records and entered into a database included leukocyte counts, C-reactive protein (CRP) level, procalcitonin level, *Candida* score, site of infection, comorbidities, epidemiological setting at the time of blood culture, severity of illness, presence of severe sepsis or septic shock, and 14-day mortality. The severity of infection was assessed using the Pitt bacteremia score at the time of blood culture [11]. The severity of comorbid conditions was assessed using McCabe classification [12].

Candidemia was defined as a minimum of one candida-positive blood culture in patients with SIRS. The time of candidemia onset was defined as the time of sampling for the first positive blood culture. The time to positivity (TTP) was determined as the time interval between the start of incubation and the detection of yeast in blood, as documented using an automated monitoring system. The time to antifungal therapy (TAT) was described as the hours elapsed between the first blood culture sample obtained and antifungal agent administration. The candidemia clearance period was defined as the duration from the first candidemia-positive blood culture to negative conversion.

*Candida* colonization was considered unifocal when *Candida* species were isolated from one focus and multifocal when *Candida* species were isolated simultaneously from various non-contiguous foci. The *Candida* score (CS) for a cut-off value of 3 was as follows: total parenteral nutrition × 1, plus surgery × 1, plus multifocal *Candida* colonization × 1, plus severe sepsis × 2 [3].

SIRS was defined based on the presence of two or more of the following conditions: body temperature > 38 °C or < 36 °C; heart rate > 90 beats/min; respiratory rate > 20 breaths/min or PaCo_2_ < 32 mmHg; and WBC > 1200 cells/mm, < 4000 cells/mm, or > 10% immature forms. Severe sepsis was defined as one or more clinical signs of organ dysfunction. Severe sepsis, septic shock, and infection type were defined according to standard criteria [13, 14]. Day 1 (D1) was defined as the first day of blood culture collection, with initial blood cultures collected within 24 h of SIRS onset in study patients. DNI_D1 and DNI_48 were defined as the DNI measured on the initial blood culture date and 2–3 days later, respectively. The site of infection was determined by physicians based on clinical evaluation. Steroid use was defined as daily use of at least 20 mg of prednisone for at least 2 weeks. Steroid administration in patients with SIRS was defined as the administration of steroids for more than 2 weeks within 3 weeks of showing signs and symptoms of SIRS. Patients with immunosuppression included those who were on immunosuppressive therapy (chemotherapeutic agents, immunosuppressive agents, or radiation therapy) within 2 weeks of showing SIRS.

### Statistical analysis

Normally distributed continuous variables are reported as means ± standard deviation (SD) and compared using Student’s t-tests. Non-normally distributed continuous variables are reported as medians with interquartile ranges (IQRs) and compared using Mann–Whitney U-tests. Categorical variables are reported as percentages and compared using chi-squared or Fisher’s exact tests, as appropriate. To measure the sensitivity and specificity of DNI values at different cutoffs, a conventional receiver operating characteristic (ROC) curve was generated and the area under the curve (AUC) was calculated to quantify the accuracy of DNI values as predictor and prognostic markers for candidemia. The cutoff values were selected to maximize the sensitivity and specificity of the DNI values. An AUC of 0.5 was considered to be no better than expected by chance, whereas a value of 1.0 signified a perfect marker. Spearman’s method was used to analyze the correlations between the time of antifungal therapy and 14-day mortality and between other factors.

Univariate and multivariate multiple logistic regression analyses were conducted to assess predictors of candidemia and prognostic factors for 14-day mortality. Variables with *p* < 0.05 in the univariate analyses and clinically significant factors were included in the multivariate analysis. Odds ratios (ORs) and their 95% confidence intervals (CIs) were calculated. All reported *p-*values were two-tailed, and *p* < 0.05 was considered statistically significant. All statistical analyses were performed using IBM SPSS Statistics for Windows, version 24.0 (IMB Corp., Armonk, NY, USA).

## Results

### Patient characteristics

The baseline clinical characteristics of the 140 patients included in the study are summarized in Table 1. The mean patient age was 65.98 ± 14.63 years and no significant difference in age was observed between the two groups. Following matching, there was no significant difference in patient locations at the time of SIRS occurrence between the case and control groups. At SIRS onset in both groups, 27 (38.6%) and 43 (61.4%) patients were in the ICU and general ward, respectively.

**Table 1:** Clinical characteristics of 140 patients with and without candidemia

	With Candidemia *N* = 70 (%)	Without Candidemia *N* = 70 (%)	Total *N* = 140 (%)	*p*
Age (mean ± SD)	67.04 ± 14.58	64.91 ± 14.71	65.98 ± 14.63	0.391
Sex				
Female	30 (42.9)	32 (45.7)	62 (44.3)	0.734
Male	40 (57.1)	38 (54.3)	78 (55.7)	
MaCabe classification				0.007
Non-fatal	13 (18.6)	27 (38.6)	40 (28.6)	
Ultimately fatal	53 (75.7)	43 (61.4)	96 (68.6)	
Rapidly fatal	4 (5.7)	0 (0.0)	4 (2.9)	
Immunosuppressant	7 (10.0)	1 (1.4)	8 (5.7)	0.063
Use of steroid	2 (2.9)	1 (1.4)	3 (2.1)	1.000
Underlying diseases				
Solid cancer	27 (38.6)	32 (45.7)	59 (42.1)	0.392
Neurologic diseases	32 (45.7)	20 (28.6)	52 (37.1)	0.036
Diabetes mellitus	22 (31.4)	22 (31.4)	44 (31.4)	1.000
Cardiovascular diseases	16 (22.9)	8 (11.4)	24 (17.1)	0.073
Chronic kidney disease	8 (11.4)	7 (10.0)	15 (10.7)	0.785
Chronic liver disease	7 (10.5)	8 (11.4)	15 (10.7)	1.000
Chronic lung diseases	2 (2.9)	5 (7.1)	7 (5.0)	0.245
Lymphoma	4 (5.7)	0 (0.0)	4 (2.9)	0.120
Solid organ transplantation	1 (1.4)	1 (1.4)	2 (1.4)	1.000
Site of infection				
Catheter related infection	48 (68.6)	0 (0.0)	48 (34.3)	< 0.001
Pneumonia	0 (0.0)	31 (42.9)	31 (22.1)	< 0.001
Primary bacteremia	20 (28.6)	0 (0.0)	20 (14.3)	< 0.001
Intra-abdominal infection	1 (1.4)	12 (17.1)	13 (9.3)	0.002
Skin and soft tissue infection	0 (0.0)	13 (18.6)	13 (9.3)	< 0.001
Urinary tract infection	1 (1.4)	8 (11.4)	9 (6.4)	0.033
CNS infection	0 (0.0)	1 (1.4)	1 (0.7)	1.000
Non-infectious SIRS	0 (0.0)	5 (7.1)	5 (3.6)	0.058
Severity of infection				
Pitt bacteremia score (median, IQR)	0 (0.0–3.0)	0 (0.0–2.0)	0 (0.0–3.0)	0.561
Severe sepsis	9 (12.9)	5 (7.1)	14 (10.0)	0.485
Septic shock	18 (25.7)	17 (24.3)	35 (25.0)	
Total parenteral nutrition	25 (35.7)	17 (24.3)	42 (30.0)	0.140
Operation	20 (28.6)	37 (52.9)	57 (40.7)	0.003
Candia colonization	19 (27.1)	6 (8.6)	25 (17.9)	0.004

*SD* Standard deviation, *IQR* Interquartile ranges

Among all patients included in this study, the most common underlying disease was solid cancer, followed by neurological disorders and diabetes. Assessment of co-morbidity severity using the McCabe classification showed a greater number of rapidly fatal cases in the case group than in the control group (5.7% vs. 0%, *p* = 0.007). There was also a clear difference in the cause of infection between the two groups (Table 1). The most common cause of candidemia in the case group was catheter-related infection (CRI), which accounted for 48 of the 70 cases (68.6%). CRIs were followed by primary bacteremia (28.6%, 20/70 cases). In contrast, the major cause of SIRS in the control group was pneumonia, which accounted for 42.9% (31/70) of the cases. However, the severity of infection assessed by the Pitt bacteremia score, severe sepsis, or septic shock did not differ significantly between the two groups (Table 1). To measure the CS, the presence of *Candida* colonization, total parenteral nutrition (TPN), and receipt of surgery were examined. The proportion of patients with *Candida* colonization and the proportion of patients who underwent surgery while hospitalized were significantly higher in the case group than in the control group (Table 1). All patients were administered antibiotics at the time of participation in this study. The overall 14-day mortality was 10.7% (15/140) and was significantly higher in the case group than in the control group (18.6% vs. 2.9%, *p* = 0.005).

### Comparison of DNI and other indicators as predictive markers of candidemia

To evaluate DNI as a predictive factor for candidemia, DNI, CRP level, procalcitonin level, leukocyte count, and CS > 3 were compared between the case and control groups (Table 2). The DNI_D1 in the case group was significantly higher than that in the control group (3.5%, [0.5–3.3] vs. 1.3% [0.1–2.4], *p* < 0.001). The DNI_48 value was also significantly higher in the case group (2.0%) than in the control group (1.0%) (*p* < 0.001). The procalcitonin level was also higher in the case group than in the control group (Table 2). However, CS > 3, a known predictive factor for candidemia, did not differ significantly between the two groups.

**Table 2:** Comparison of delta neutrophil index to other laboratory markers for predicting candidemia

Laboratory marker	With Candidemia *N* = 70 (%)	Without Candidemia *N* = 70 (%)	Total *N* = 140 (%)	*p*
DNI (median, IQR)	3.5 (2.3–5.4)	1.3 (0.1–2.4)	2.4 (0.9–4.0)	< 0.001
DNI_48 (median, IQR)	2.0 (0.5–3.3)	1.0 (0.0–2.3)	1.3 (1.0–2.6)	0.004
Leukocytes, 10^3^/uL (mean ± SD)	11,087.6 ± 5898.8	9379.6 ± 3808.2	10,079.1 ± 5216.5	0.081
CRP, mg/L (median, IQR)	82.0 (50.3–144)	66.8 (29.6–10.5.8)	74.5 (38.0–132.3)	0.249
Procalcitonin, mg/dL (median, IQR)	0.72 (0.29–1.91)	0.34 (0.18–0.66)	0.42 (0.19–1.43)	0.025
Candia Score ≥ 3	17 (24.3)	18 (25.7)	35 (25.0)	0.845

*DNI* delta neutrophil index, *IQR* Interquartile ranges, *SD* Standard deviation, *CRP* C-reactive protein

As our control group included five non-infectious SIRS patients, we also compared the DNI values between patients with clinically documented infections and non-infectious SIRS in the control group. The DNI_D1 values were 1.3% (0.2–2.4) in patients with clinically documented infection and 0.0% (0.0–1.0) in those with non-infectious SIRS. While the patients with clinically documented infection tended to show higher DNI_D1 value, the difference was not statistically significant (*p* = 0.304). A similar trend was observed for DNI_48 values (1.0% vs. 0.2%, *p* = 0.178). Comparison of the case and control groups after excluding patients with non-infectious SIRS showed that the clinical characteristics did not differ from those of the entire control group. The DNI_D1 value in the case group (3.5%, 0.5–3.3) was significantly higher than that in the control group after excluding patients with non-infectious SIRS (1.3%, 0.2–2.4) (*p* < 0.001). The DNI_48 value was also significantly higher in the case group (2.0%) than in the control group after excluding patients with non-infectious SIRS (1.0%) (*p =* 0.010).

The results of multivariate analyses to determine the independent predictive factors for candidemia in the case and control groups are summarized in Table 3. The factors that differed significantly in univariate analysis, CS > 3 used for predicting candidemia, and clinically important factors exhibiting insignificant differences in univariate analysis were included in the multivariate analysis.

**Table 3:** Multivariate analyses evaluating the predictive factors of candidemia

	With Candidemia *N* = 70 (%)	Without Candidemia *N* = 70 (%)	ORs (95% CI)	*p*
DNI_D1, % (median, IQR)	3.5 (2.3–5.4)	1.3 (0.1–2.4)	2.138 (1.421–3.217)	< 0.001
DNI_48, % (median, IQR)	2.0 (0.5–3.3)	1.0 (0.0–2.3)	0.913 (0.713–1.169)	0.471
Leukocytes, 10^3^/uL (mean ± SD)	11,087.6 ± 5898.8	9379.6 ± 3808.2	1.000 (1.000–1.000)	0.238
CRP (median, IQR)	82.0 (50.3–144)	66.8 (29.6–10.5.8)	1.001 (0.993–1.010)	0.729
Procalcitonin (median, IQR)	0.72 (0.29–1.91)	0.34 (0.18–0.66)	1.082 (0.775–10,512)	0.643
McCabe classification Rapidly fatal	4 (5.7)	0 (0.0)	2.013 (0.574–7.055)	0.275
Operation	20 (28.6)	37 (52.9)	0.973 (0.330–2.872)	0.961
Candida colonization	19 (27.1)	6 (8.6)	7.361 (1.717–31.553)	0.007
Neurologic disease	32 (45.7)	20 (28.6)	1.683 (0.561–4.782)	0.367

*DNI* Delta neutrophil index, *IQR* Interquartile ranges, *SD* Standard deviation, *CRP* C-reactive protein

The multivariate analyses identified DNI_D1 (OR, 2.183, 95% CI, 1.421–3.217, *p* < 0.001) and *Candida* colonization (OR, 7.361, 95% CI, 1.717–31.553, *p* = 0.007) as useful indices for predicting candidemia (Table 3). Multivariate analyses of the control group, including only patients with clinically documented infection, also identified DNI_D1 (OR, 2.139, 95% CI, 1.424–3.213, *p* < 0.001) and *Candida* colonization (OR, 7.638, 95% CI, 1.732–33.676, *p* = 0.007) as useful indicators for predicting candidemia.

### Optimal cutoff value of DNI for predicting candidemia

**Fig. 1:**
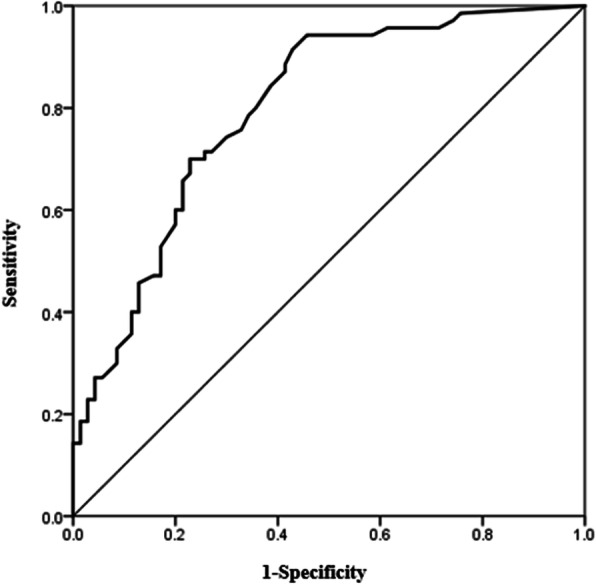
Receiver operating characteristics (ROC) curve to determine the cutoff value of delta neutrophil index (DNI) for predicting candidemia in patients with systemic inflammatory syndrome. Area under ROC was 0.804 (95% CU, 0.719–0.890)

ROC curve and AUC analyses were conducted to determine the DNI_D1 cutoff value for predicting candidemia (Fig. 1). The optimal cutoff value was 2.75% and the AUC of DNI_D1 was 0.804 (95% CI, 0.719–0.890, *p* < 0.001). The sensitivity and specificity in predicting candidemia for a cutoff DNI value of 2.75% were 72.9 and 78.6%, respectively, and the positive and negative predictive values were 77.3 and 74.3%, respectively. For DNI_D1 > 2.75%, the OR for the presence of candidemia was 9.842 (95% CI, 4.562–21.402, *p* < 0.001).

In comparison with the control group, including only patients with clinically documented infection, the cutoff value was 2.75%, and the AUC of DNI_D1 was 0.796 (95% CI, 0.721–0.871, *p* < 0.001). The sensitivity, specificity, positive predictive, and negative predictive value were 72.9, 76.9, 77.3, and 72.5%, respectively. The OR for the predicting candidemia was 8.947 (95% CI, 4.096–19.544, *p* < 0.001).

### Factors associated with 14-day mortality in patients with candidemia

To determine the prognostic factors for mortality in patients with candidemia, various factors were comparatively analyzed according to 14-day mortality. Among 70 patients with candidemia, 13 died within 14 days of candidemia onset. There were no differences in age, sex, or patient location at the time of candidemia onset (*p* = 0.210) between the survivor and non-survivor groups. The underlying diseases and site of infection also did not differ significantly between the survivor and non-survivor groups. The non-survivor group showed a significantly higher Pitt bacteremia score (4.0 [1.0–5.0] vs. 0.0 [0.0–2.0], *p* < 0.001) and septic shock rate (61.5% vs. 7.5%, *p* = 0.003) than those in the survivor group. The percentage of patients with CS ≥3 was higher in the non-survivor group than in the survivor group (46.2% vs. 19.3%, *p* = 0.042). DNI_D1 and DNI_48 values were also significantly higher in the non-survivor group than in the survivor group (7.4% [4.0–22.0] and 6.1% [2.1–14.4], respectively. The TTP in all patients with candidemia was 48 h (32.0–73.0, range 10–120 h). Among patients with candidemia, the most common *Candida* species was *C. albicans* (33/70, 47.1%) followed by *C. tropicalis* (16/70, 22.9%), *C. parasilopsis* (10/70, 14.3%), and *C. glabrata* (9/70, 12.7%). The TTPs for the four most frequently identified *Candida* species were 68 h (47.0–78.0), 30.5 h (26.5–33.5), 47.0 h (34.0–58.0), and 72.0 h (50.5–93.0), respectively.

The TTP in the non-survivor group was shorter than that in the survivor group, but the difference was not statistically significant (31.0 h [26.5–53.0] vs. 48.0 h [34.0–72.0], *p* = 0.066).

The non-survivor group showed a significantly shorter TAT than did the survivor group (36 h [12.5–42.0] vs. 60 h [42.0–96.0] *p* = 0.013), showing that antifungal therapies were administered earlier to the non-survivor group. As shown in Table 4, no significant difference was observed in TAT between the survivor and non-survivor groups in the multivariate analysis. However, antifungal therapy tended to be administered faster, with earlier *Candida* detection from blood cultures (*r* = 0.56 *p* = 0.044). In addition, TAT and 14-day mortality showed an inverse correlation (*r* = − 0.33, *p* = 0.011).

**Table 4:** Comparisons of prognostic factors of 14-day mortality between surviving and non-surviving patients with candidemia

	Survivor *N* = 57 (%)	Non-survivor *N* = 13 (%)	*p*	Adjusted ORs (95% CI)	*p*
Age (mean ± SD)	65.81 ± 13.361	72.46 ± 18.715	0.959		
Sex					
Female	27 (47.4)	3 (23.1)	0.110		
Male	30 (57.1)	10 (76.9)			
Co-morbidities					
MaCabe classification			0.163		
Non-fatal	12 (21.2)	1 (7.7)			
Ultimately fatal	43 (75.4)	10 (76.9)			
Rapidly fatal	2 (3.5)	2 (15.4)			
Severity of infection					
Pitt bacteremia score (median, IQR)	0 (0.0–2.0)	4.0 (1.0–5.0)	< 0.001	1.187 (0.890–1.584)	0.244
Septic shock	10 (17.5)	8 (61.5)	0.003	7.635 (1.159–50.290)	0.035
Candia colonization	15 (26.3)	4 (30.8)	0.739		
Candida score ≥ 3	11 (19.3)	6 (46.2)	0.042	0.900 (0.225–3.609)	0.882
DNI_D1, %	3.4 (2.2–5.3)	7.4 (4.0–22.0)	0.002	1.156 (1.039–1.287)	0.008
DNI_48, %	2.0 (0.5–2.9)	6.1 (2.1–14.4)	0.015	1.226 (1.007–1.494)	0.043
Leukocytes, 10^3^/uL (mean ± SD)	10,831.58 ± 6010.190	10,931 ± 7311.871	0.139		
CRP (median, IQR)	66.0 (39.0–145.0)	115.5 (84.2–136.0)	0.036	1.003 (0.996–1.009)	0.412
Procalcitonin (median, IQR)	0.47 (0.2–1.9)	1.0 (0.38–2.09)	0.303		
Time to positive, hours, (median, IQR)	48.0 (34.0–72.0)	31.0 (26.5–53.0)	0.066		
Time to antifungal therapy, hours (median, IQR)	60 (42.0–96.0)	36 (12.5–42.0)	0.013	0.968 (0.933–1.004)	0.079
Duration of Clear- up, days, (median, IQR)	5 (3.0–9.5)	4.5 (4.0–5.0)	0.638		

*DNI* Delta neutrophil index, *IQR* Interquartile ranges, *SD* Standard deviation, *CRP* C-reactive protein

The duration until the negative conversion of candidemia also did not differ between the survivor and non-survivor groups (Table 4).

Multivariate analysis also confirmed that DNI_D1 (OR, 1.156, 95% CI, 1.039–1.287, *p* = 0.008) and the occurrence of septic shock were reliable prognosis factors for 14-day mortality. The DNI_D1 cutoff value for predicting 14-day mortality was 3.95%, and the AUC was 0.769 (95% CI, 0.624–0.914, *p* = 0.001).

## Discussion

The results of this study confirmed the DNI to be a predictive marker of candidemia in patients with SIRS and a prognostic marker for patients with candidemia.

Candidemia is associated with one of the highest rates of mortality of any bloodstream infection (BSI) [15]. To reduce mortality due to candidemia, AAT needs to be administered as early as possible. The current initiation of antifungal therapy upon identification of the yeast from the blood culture results in delayed treatment. However, avoiding delays in the treatment of patients with candidemia is difficult. As the risk factors currently known to be associated with *Candidemia* are also related to drug-resistant bacterial infection, they do not help differentiate candidemia from a bacterial infection in patients manifesting SIRS [16]. Various scoring systems and markers have been developed to distinguish candidemia, such as the CS or *Candida* colonization index [3–5]. However, their sensitivity for differentiating invasive *Candida* infection and colonization is only around 60% [6]. As markers such as Β-D-glucan are generally not used by all hospitals, they have limits in their application. The administration of empirical antifungal therapy can be considered in patients suspected of sepsis but can cause problems like inappropriate administration of antifungal therapy and increased resistance.

The DNI is a novel index reflecting a circulating fraction of immature granulocytes (IGs) [17, 18]. Previous studies identified the DNI as a factor that distinguishes infection from non-infection and a prognostic factor for severity in patients with sepsis [19–22]. Although studies on the differentiation between bacterial infection and non-infectious condition have been reported, there are no studies on invasive *Candida* infection, candidemia prediction, or candidemia prognosis assessment. The DNI can be calculated automatically while measuring CBC. The CBC is routinely and frequently evaluated in patients with SIRS at a substantially lower cost than other laboratory markers. The DNI can be easily calculated and reported without an additional cost. Using samples collected from patients at SIRS onset in our study of 70 patients with candidemia and 70 patients without candidemia, a DNI_D1 > 2.75% had a sensitivity of 72.9% and a specificity of 78.6% for predicting candidemia. In SIRS patients, *Candida* colonization was also identified as a predictor of candidemia, along with the DNI_D1. The cause of infection was mostly CRI or primary bacteremia in patients with candidemia. Among patients with suspected SIRS and sepsis, empirical antifungal therapy can be restrictively considered for patients with *Candida* colonization and potential CRI or primary bacteremia due to the absence of clear infection site based on a DNI cutoff of 2.75%. In this study, the median *Candida* detection time was 48 h (32–73). The preemptive administration of antifungal therapy only to selected patients may permit economical early administration of antifungal therapy compared to the administration of empirical antifungal agents to all patients. Based on the results of previous studies reporting that short-term fluconazole administration was not associated with increased fluconazole resistance in patients with candidemia [23, 24], preemptive antifungal therapy can be administered to patients with SIRS, *Candida* colonization, ambiguous primary infection sites, and increased DNI values. Alternatively, in patients with SIRS with DNI < 2.75% and clinically clear sites of infection, we may consider discontinuing empirical antifungal therapy. Using DNI in this way contributes to antifungal stewardship in empirical antifungal therapy.

DNI_D1 and DNI_48 were identified as independent, predicting factors for 14-day mortality in patients with candidemia. Many patients in the non-survivor group also had septic shock. Although it is generally known that mortality can be reduced if antifungal therapy is administered earlier, the results of this study demonstrated earlier administration of antifungal therapy in the non-survivor group compared to that in the survivor group (36 h [12.5–42.0] vs. 60 h [42.0–96.0]). TTP was also administered earlier in the non-survivor group than in the survivor group. As previously reported, the TTP of blood culture is associated with the microbial burden in the blood [25, 26].. In other words, a shorter TTP indicates a higher microbial burden. The shorter TTP (36 h vs. 60 h, *p* = 0.066) in the non-survivor group suggested a higher *Candida* burden in the non-survivor group, thus leading to severe infection. Also, higher DNI values in the non-survivor group suggested that the DNI reflected the severity of candidemia. As in the case of bacterial infection, increased infection severity leads to increased DNI values [8, 19, 21].

To date, antifungal therapy for candidemia generally begins when yeast is detected in the blood culture. This explains why antifungal therapy began earlier in the non-survivor group in this study. In the non-survivor group, the median TAT and TTP were 36 and 31 h respectively, suggesting antifungal therapy was initiated after the detection of yeast from the blood culture. Earlier administration of antifungal therapy based on the DNI may increase the survival rate of candidemia patients, especially those with severe sepsis or septic shock.

This study has several limitations. First, this study was performed at a single center and the results were analyzed retrospectively. Thus, despite matching, selection bias is possible. Second, DNI elevation is not specific to infection and may be observed in various other conditions, including hematologic malignancy, acute hemorrhage, and chronic inflammatory diseases [18]. Third, this study classified patients with candidemia into the case group and patients without bacteremia into the control group. We cannot exclude the possibility that these selection criteria might contribute to differences in DNI values between the two groups. For these reasons, further prospective analyses with larger populations are needed to confirm the DNI as a prediction and prognostic marker of candidemia.

## Conclusions

The results of this study confirmed the DNI as a predictive and prognosis factor for candidemia in patients with SIRS. With recent advancements in testing tools, the DNI can be obtained easily and quickly. Although prospective studies with larger numbers of patients are needed, the ability to use DNI values and clinical characteristics (*Candida* colonization or site of infection) to screen patients at high risk for candidemia in real clinical settings may contribute to a decrease in candidemia mortality.

## Data Availability

The datasets used and analyzed during the current study are publicly available from the corresponding author on reasonable request and permission of the Institutional Review Board of Kangdong Sacred Heart Hospital in Seoul, South Korea.

## Abbreviations

AAT: Appropriate antifungal therapy
DNI: Delta neutrophil index
SIRS: Systemic inflammatory response syndrome
ICU: Intensive care units
IRB: Institutional Review Board
CBC: Complete blood count
WBC: White blood cell
MPO: Myeloperoxidase
PMN: Polymorphonuclear neutrophil
CRP: C-reactive protein
TTP: Time to positivity
TAT: Time to antifungal therapy
CS: *Candida* score
SD: Standard deviation
IQR: Interquartile range
ROC: Receiver operating characteristic
AUC: Area under the curve
ORs: Odds ratio
CI: Confidence interval
IG: Immature granulocyte
